# Transmission time delays organize the brain network synchronization

**DOI:** 10.1098/rsta.2018.0132

**Published:** 2019-07-22

**Authors:** Spase Petkoski, Viktor K. Jirsa

**Affiliations:** Institut de Neurosciences des Systèmes (INS), Inserm, Aix Marseille Univ, Marseille, France

**Keywords:** synchronization, time delays, connectome, brain network model, oscillators

## Abstract

The timing of activity across brain regions can be described by its phases for oscillatory processes, and is of crucial importance for brain functioning. The structure of the brain constrains its dynamics through the delays due to propagation and the strengths of the white matter tracts. We use self-sustained delay-coupled, non-isochronous, nonlinearly damped and chaotic oscillators to study how spatio-temporal organization of the brain governs phase lags between the coherent activity of its regions. *In silico* results for the brain network model demonstrate a robust switching from in- to anti-phase synchronization by increasing the frequency, with a consistent lagging of the stronger connected regions. Relative phases are well predicted by an earlier analysis of Kuramoto oscillators, confirming the spatial heterogeneity of time delays as a crucial mechanism in shaping the functional brain architecture. Increased frequency and coupling are also shown to distort the oscillators by decreasing their amplitude, and stronger regions have lower, but more synchronized activity. These results indicate specific features in the phase relationships within the brain that need to hold for a wide range of local oscillatory dynamics, given that the time delays of the connectome are proportional to the lengths of the structural pathways.

This article is part of the theme issue ‘Nonlinear dynamics of delay systems’.

## 1. Introduction

Rhythms are ubiquitous among dynamical systems, many of which are connected in complex structures, such as the brain. Functionally relevant brain oscillations span several orders of magnitude in frequency [1], and coherent oscillations between distant regions that occur during cognitive tasks [2–4] have been proposed as a mechanism for information transfer [5] and routing [6] in the brain. As with other interacting oscillatory systems, coherent brain rhythms [7] are often a hallmark of synchronization [8], and for large-scale brain dynamics they describe the functional connectivity of the brain [9–11], which is confined by its structure [12–15], i.e. the so-called connectome [16]. Neural processing and communication are in this way fundamentally regulated by the couplings between distant brain regions, which shape the spatio-temporal organization of the brain through links' strengths and time delays due to propagation [17–19]. Many groups have thus tried to link the interpretation of neuroimaging signals to computational brain models for healthy [20–23] or pathological [24,25] brain activity, often employing neuroinformatics platforms such as The Virtual Brain (see http://www.thevirtualbrain.org) [26,27].

Phase relationships offer a good insight into functional interactions between oscillatory processes across the brain [19,28], but are mainly overlooked when studying the synchronization in complex networks [29], or more specifically in the brain [3,14,30]. For the latter, heterogeneous delays have been shown to be of critical importance for the observed in- and anti-correlations [31,32], or for the phase relationship between local node dynamics and their degree [28,33].

Dynamics of networks with spatially distributed delays have been recently analysed [18,19,28], but only for reductions to phase oscillators with sine coupling [34]. Oscillatory processes are particularly sensitive to delays, and for coupled multi-dimensional oscillators, time delays are known to cause amplitude and oscillation death [35]. They also facilitate synchronization of spike-burst networks [36], the control of chimeras [37], and lead to enhanced [38–40] and zero-lag synchronization [41] in brain and behaviour [42].

The aim of this work is to describe the impact that the heterogeneous time delays of the brain network have over its emergent activity, given that the mesoscopic governing dynamics are oscillatory [1,3]. These are often described by the normal form of Andronov-Hopf (AF) bifurcation [28,43,44], which also encompasses the working points of population rate models [21,31]. To widen the scope, besides two realizations of the supercritical AH bifurcation: a Landau–Stuart (LS) with amplitude dependent phase, and a van der Pol (VdP) limit cycle with a nonlinear damping [45], we also analyse a chaotic neural dynamics represented by a Rössler oscillator [46]. Synchronization of the *in silico* BNM neural activity generally confirms the patterns of phases in networks of Kuramoto oscillators with heterogeneous delays [18,19], where the analytical results for bimodal structured delays were verified for the connectome [19]. Namely, brain hemispheres, which can be approximated as delay-defined clusters [18], were shown to intermittently switch from in- to anti-phase synchronization for higher frequencies and small couplings, with stronger nodes generally lagging in phase [19]. The richer local oscillatory dynamics applied here makes the anti-phase synchronization more robust, and also gives insight into the impact of the delays on the amplitude of oscillations, which is shown to decrease for more synchronized brain regions by increasing the frequency. Thus, we identify common network manifestations of the time delays for a range of self-sustained oscillatory neural activity, beyond the already treated cases [18,19,28] that allow reduction to the KM [34] due to the disentanglement of the amplitude and the phase.

## 2. Model and methods

### (a) Brain network model

The model is built over connectome-based architecture that dictates the strength and the delay of the interactions between brain areas, whose inherent averaged neural activity is described with three types of self-sustained amplitude oscillators.

**Figure 1.**
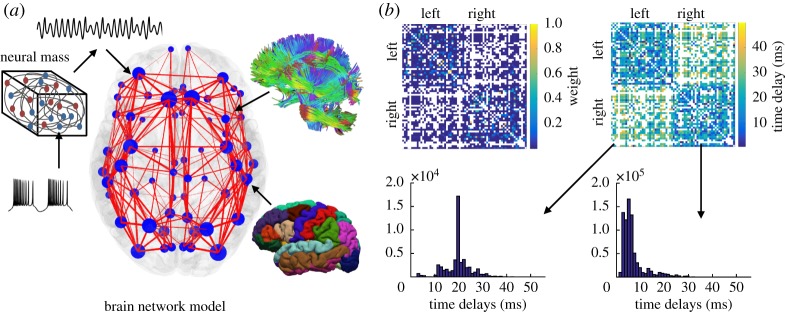
(*a*) Brain Network Model consisting of the neural mass dynamics superimposed over the connectome, which is reconstructed using DTI tractography (top) and MRI topography (bottom). The width of the links (red lines) and the size of the brain regions (blue circles) are proportional to their strengths. (*b*) (top) Matrices of the strengths and time delays of the links between different regions, and (bottom) distribution of weighted inter- (left) and intra-hemispheric (right) delays.

A healthy human connectome is chosen from the Human Connectome project [47], where a customized 3 *T* scanner was used for the magnetic resonance imaging (MRI). The structural connectivity is reconstructed from the diffusion tensor imaging (DTI) using a pipeline [48–50] that yields *N* = 68 cortical regions, delineated according to the Desikan–Kiliany atlas [51], figure 1*a*. For each link, its weight is the numbers of individual tracts between the pair of regions, and their mean length divided by the propagation velocity that is set at 5 m s^−1^, which is within the experimental range [52], gives the time delay associated with that link, figure 1*b*.

For all three types of oscillators, there is a variable resembling the natural frequency and it is set to *ω*_0_ = 1 rad s^−1^ for all the nodes. The chaotic system has the form

2.1 $$\begin{array}{rl} & {\dot{x}}_{i}=-\omega_{0}y_{i}-z_{i}+\eta_{i}(t)+\frac{1}{N}\sum_{j=1}^{N}K_{ij}[x_{j}(t-\tau_{ij})-x_{i}]\\ & {\dot{y}}_{i}=\omega_{0}x_{i}+ay_{i}\\ \mathrm{and}\; & {\dot{z}}_{i}=b+z_{i}(x_{i}-c),\;i=1\ldots N, \end{array}\}$$

where parameters are set to be: *a* = 0.2, *b* = 0.2 and *c* = 5.7, so that untrained oscillators are in chaotic regime. The nonlineary damped oscillator has the form

2.2 $$\begin{array}{rl} & {\dot{x}}_{i}=2m(1-\beta y^{2})x-\omega_{0}^{2}x+\eta_{i}(t)+\frac{1}{N}\sum_{j=1}^{N}K_{ij}[x_{j}(t-\tau_{ij})-x_{i}]\\ \mathrm{and}\; & {\dot{y}}_{i}=x_{i},\;i=1\ldots N, \end{array}\}$$

where the nonlinearity is controlled with the term *m* that is set to 0.75, with *m* = 0 corresponding to harmonic oscillator, and *m*≪1 being the quasi-linear case. The non-isochronous oscillator has the form

2.3 $${\dot{X}}_{i}=X_{i}[(1+j\omega_{0})-(1+jq)|X{|}^{2}]+\eta_{i}(t)+\frac{1}{N}\sum_{j=1}^{N}K_{ij}[X_{j}(t-\tau_{ij})-X_{i}],\;i=1\ldots N,$$

where *X*_*i*_ = *x*_*i*_ + *jy*_*i*_, with *j* representing an imaginary unit, the amplitude is set to 1, and the level of non-isochronicity is set to *q* = 0.5, with *q* = 0 corresponding to the isochronous case when the phases and the amplitudes are untangled. For each link, *K*_*ij*_ and *τ*_*ij*_ are coupling strengths and time delays, whereas for the additive Gaussian noise 〈*η*_*i*_(*t*)〉 = 0 and 〈*η*_*i*_(*t*)*η*_*j*_(*t*′)〉 = 2*Dδ*(*t* − *t*′)*δ*_*i*,*j*_, with 〈 · 〉 denoting time-average operator.

The coupling takes the form of a linear difference, as the simplest approximation of the general coupling function, and it affects the first variables for the Rössler and VdP systems, and both variables for the LS oscillators, as is usually the case [8]. Choosing a linear additive coupling would allow obtaining the same form by rescaling the parameters of the general AF bifurcation in its realizations as LS and VdP oscillators. For the Rössler case, the difference coupling is not transformable to the additive one, and even though *y* and *x* are linearly interdependent, indicating that a rescaling should be possible in order to obtain qualitatively similar dynamics, this is not as simple as for the LS and VdP oscillators. It is worth noting that even biologically inspired neural mass models where chemical synapses lead to much more complex interactions often operate close to the AF bifurcation [21,31], allowing linearization of the coupling function. Nevertheless, an analysis of different working points of these models might also be required to fully understand the impact of the delays for the large-scale brain dynamics.

The principal component of the oscillations in each model is set to the chosen frequency for the simulated neural activity by rescaling the time. This is possible because the models are phenomenological and the parameters are chosen such that they would preserve the chaotic, nonlinear and non-isochronous regimes respectively. We fix the velocity of propagation in the connectome at 5 m s^−1^, which is within the experimental range [52], and following the earlier work [19], we choose frequencies of 5 Hz and 20 Hz as values that are expected to show distinctive dynamics due to the time delays. As the natural frequencies are set at *ω*_0_ = 1 rad s^−1^, the time is accordingly rescaled with factors 5 × 2*π* and 20 × 2*π*, while the mean intra- and inter-hemispheric time delays are *τ*_int_ = 6.5 ms and *τ*_ext_ = 19.6 ms, respectively.

### (b) Analysis of the phase dynamics

#### (i) Phases from time-series.

The complexity of the model makes the analytical derivations of phases at each region a cumbersome, and probably impossible task. Instead, in order to quantify the phase dynamics, angle variables [8] are defined for each node as

2.4 $$\varphi_{i}=\arcsin\frac{y_{i}}{x_{i}}.$$

These represent protophases that are then used to obtain the phases [53], which by definition need to grow linearly, although this step is often skipped [8]. Alternatively, in the time-series analysis the protophase is often estimated from an oscillatory variable obtained by Hilbert or Wavelet Transform [8,54]. The phases are calculated from protophases as [53]

2.5 $$\theta_{i}=\langle \dot{\varphi_{i}}\rangle \int_{0}^{\varphi }{\left[\frac{d\varphi_{i}}{dt}\right]}^{-1}\;d\varphi_{i}.$$

Note that for all the analysis, a steady synchronization arises at frequency $\Omega =\dot{\Psi }={\dot{\theta }}_{i}{|}_{\mathrm{sync}}$ [18,19], where the mean field phase *Ψ* [55] is obtained from the complex order parameter of each hemisphere

2.6 $$re^{i\Psi }=\frac{1}{N}\sum_{j=1}^{N_{\mathrm{hem}.}}e^{i\theta_{j}},$$

where *r* defines the level of synchrony. Thus, the analysis of the phases is completed in the rotating frame *Ω* where the relative phases are rewritten as $\theta_{i}\to \theta_{i}-\Omega t$.

#### (ii) In- and anti-phase coherent network dynamics: theoretical background.

For validation of the numerical results, we use an expression that was derived for the phases of synchronized oscillators relative to the hemispheric mean fields for a BNM with Kuramoto oscillators [19]. Assuming that the synchronization of each node depends on its strength *K*_*i*_, which is defined as the sum of weights of all links connecting the node *i*, it reads

2.7 $$\langle \theta_{i}\rangle ≊\arcsin\left(\frac{\omega_{0}-\Omega }{K_{i}r\cos\Omega \Delta \tau }\right)-\Omega \tilde{\tau },$$

where Δ*τ* = (*τ*_ext_ − *τ*_int_)/2 and $\tilde{\tau }=(\tau_{\mathrm{ext}}+\tau_{\mathrm{int}})/2$ for the means of the weighted inter- (external) and intra-hemispheric (internal) delays. Anti-phase synchronization appears for *Ωτ*_ext_ in the left complex half-plane [19], and the condition for synchronization of each region reads

2.8 $$|\omega_{0}-\Omega |\leq K_{i}r\cos\Omega \Delta \tau .$$

#### (iii) Statistical analysis of the phase locking.

Phase relationships between brain regions are quantified using phase locking values (PLV) [7], which are a statistical measure for similarity between phases of two signals, frequently employed in the analysis of empirical data. Although synchronization can occur between different frequencies [54,56], most commonly studied is the one-to-one entrainment, for which the complex phase locking values (cPLV) are defined as

2.9 $${\mathrm{cPLV}}_{ij}\equiv {\mathrm{PLV}}_{ij}e^{i\phi_{ij}}=\frac{1}{M}\sum_{p=1}^{M}e^{i\Delta \theta_{ij}(p)},$$

where the phase difference Δ*θ*_*ij*_(*p*) = *θ*_*i*_(*p*) − *θ*_*j*_(*p*) is calculated at times *p* = 1…*M*. Here, we calculate cPLV at sliding windows of length equal to 5 periods of the calculated mean frequency of the entrainment, *Ω*, [55] and with 25% overlap. To identify only the phase coherence due to mutual interactions [7,19,57], we calculate a level of statistical significance for PLV as the 95th percentile of maximum values in 100 surrogate signals that are obtained by shuffling one of the phases, and which follow the same processing as the original signals.

## 3. Results

**Figure 2.**
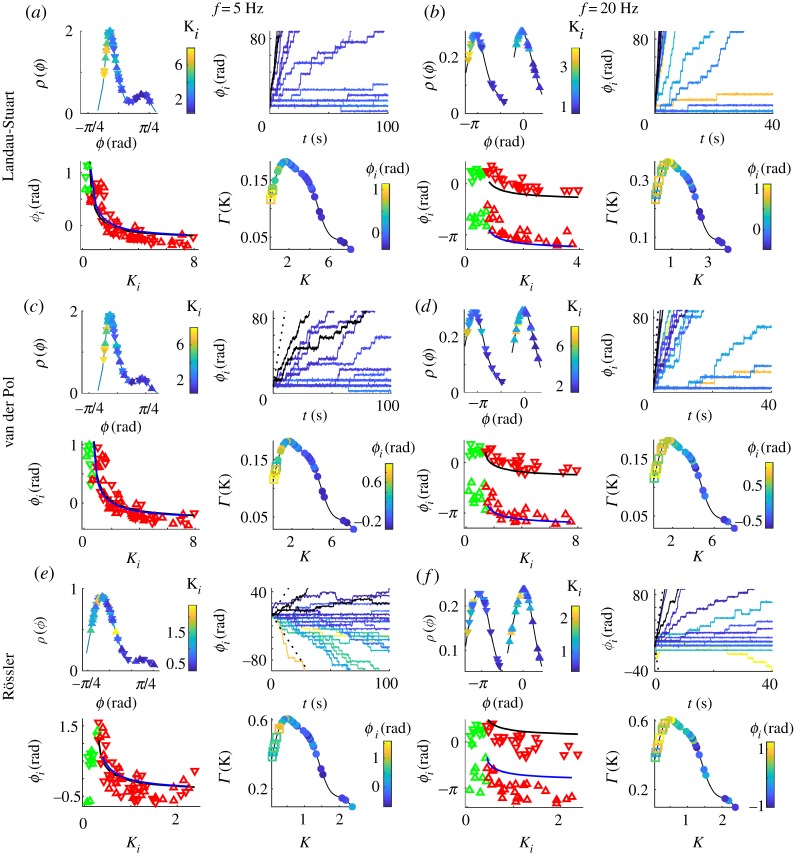
In- (5 Hz) and anti-phase (20 Hz) synchronization for simulated dynamics over a healthy human connectome. Top left plots in each panel are the phases of the synchronized regions (colour-coded with their strength) and their PDF. Top right are evolutions of phases *ϕ*_*i*_(*t*) for the synchronized (colour-coded with their strength) and two strongest unsynchronized oscillators (black), and ± (*Ω* − *ω*)*t* (dashed). Bottom left are scatter plots of averaged phases and in-strengths, with nodes of left/right hemisphere being up/down pointing triangles (red for synchronized, green otherwise), and blue and black lines being analytical predictions equation (2.7). Bottom right are the PDF of nodes strengths colour-coded with their relative phases (full circles for synchronized, and hollow squares otherwise). Parameters: noise intensity (*a*,*e*,*f* ) *D* = 0.25, (*b*,*d*) *D* = 0.01 and (*c*) *D* = 0.5, coupling strengths (*a*,*c*,*d*) *K* = 0.02, (*b*) *K* = 0.01, (*e*,*f* ) *K* = 0.006.

Even though all three oscillatory systems making up the BNM have more complex dynamics than the harmonic phase oscillators [19], they also synchronize such that brain hemispheres are in- and anti-phase locked, depending on the frequency, as in figure 2. Here, scatter plots of phases and nodes strengths are depicted, together with theirs probability density distributions (PDF) and the time-evolution of the phases in the rotating frame *Ω* of the mean-field. Left panels of figure 2 are for a frequency *f* = 5 Hz, so that even the long inter-hemispheric time delays do not cause phase shifts *Ωτ* larger than *π*/2, while in the right panels *f* = 20 Hz and the phase shift is in the left complex half-plane. As with the KM, within the hemispheres the stronger nodes generally lag in phase behind the weaker, while the *π* shift needs to be accounted for when comparing contralateral regions during anti-phase regime. Moreover, the analytical result for the relative phases, equation (2.7), holds quite well, with the only significant deviation appearing for the chaotic oscillators.

**Figure 3.**
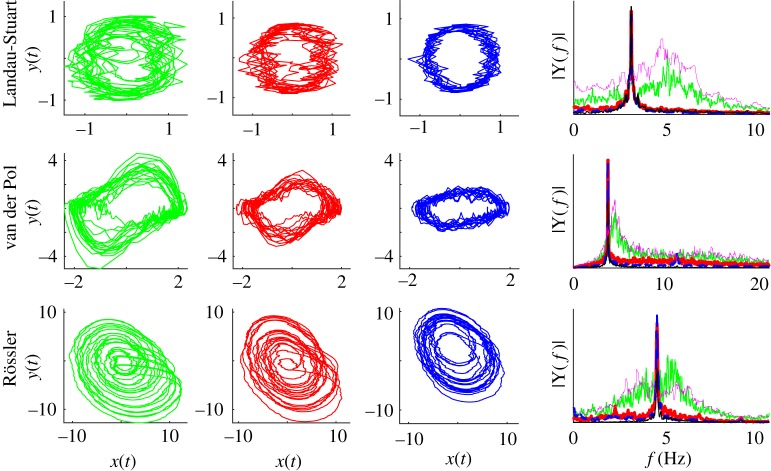
Phase portraits and Fourier spectra for LS, VdP and Rössler BNM. (left) Twenty cycles are depicted for the weakest (green), the medium (red) and the strongest (blue) connected nodes. The same colours are used for (right) the amplitude spectra of the neural activity (x-components) of the same nodes, as well as an uncoupled oscillator (magenta), and the local complex parameters (black). Parameters are the same as in figure 2 for frequency *f* = 5 Hz.

For the isochronous case of LS oscillators, their phase dynamics is fully captured by the KM [34]. However, even for the non-isochronous case chosen here, as with the nonlinear VdP, the phase shifts during different regimes of synchronization are similar to the case of KM [19]: in general, weaker nodes drift forward, and the stronger lag behind, while being locked to the mean field for most of the time. Contrary to the KM, the anti-phase synchronization is not necessarily intermittent for BNM using amplitude oscillators, and therefore the arrangement of the phases within the hemispheres is not affected by the type of locking (in- or anti-phase). The only anomalous behaviour is for small delays and chaotic oscillators, when some of the stronger connected nodes lag behind the mean field [55], which is more aligned to the weaker synchronized nodes. This slowing down is not reflected in the peak frequencies of the power spectrum, which are identical for synchronized oscillators of different strength, figure 3, and they coincide with the frequency of the complex order parameter. The power spectrum also shows that the entrainment for chaotic low-frequency activity is at a higher frequency than the natural, i.e. the frequency of an uncoupled oscillator. Nevertheless, this does not seem to reverse the dependence of the relative phases of the nodes strength, unlike for phase oscillators [19], suggesting that an artefact in the recovery of phases might be the cause of this anomaly.

**Figure 4.**
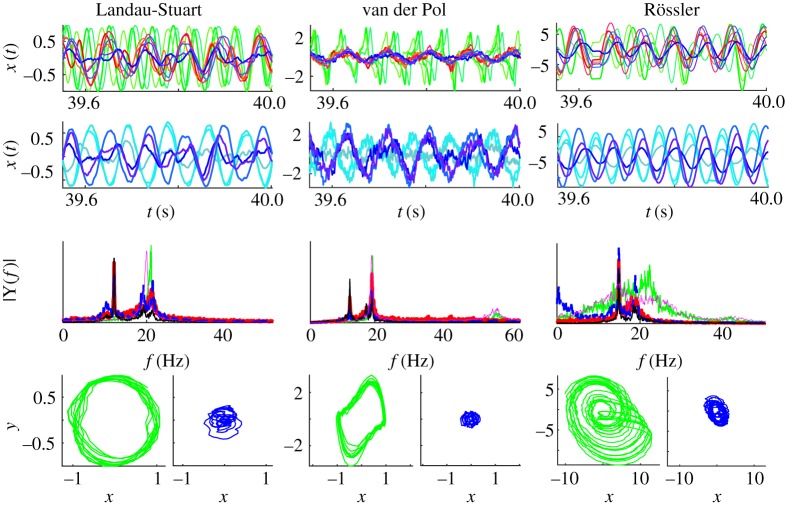
Time-series, Fourier spectra and phase portraits of LS, VdP and Rössler BNM. The x-component of the oscillators is depicted for the three strongest (shades of blue), three weakest (shades of green) and three nodes with medium strength (red) of the left hemisphere, and three strongest nodes of the right hemisphere. The same colour code is used across the plots, while the Fourier spectra also depict an uncoupled oscillator (magenta), and the local complex order parameters (black) equation (2.6). Parameters are the same as in figure 2 for frequency *f* = 20 Hz.

Phase portraits in figure 3 show that network interactions inevitably cause distortions of limit-cycles, beyond the stochastic nature of the added noise. This gets more pronounced when delays are comparable with the inherent time-scale of the oscillators, as is demonstrated in figure 4 for all three BNMs at frequency *f* = 20 Hz. The strongest nodes are mostly affected, and in general the amplitude decreases with the strength of the nodes, so the oscillations become less nonlinear and more stochastically driven. In the case of chaotic oscillators, they become more regular and harmonic, the same as for the nonlinearly damped model.

Time-series of the neural activity from nodes with a different strength, figure 4, depict the in- and anti-phase synchronization within and between the hemispheres, respectively. Three of the strongest, the weakest and medium strength nodes within the same hemisphere are shown for each of the models, as well as the activity of strongest contralateral nodes. In all the cases, the weakest nodes are unsynchronized with the rest, and the strongest and the medium nodes are rather coherent between each other, while being anti-correlated with the opposite hemisphere. This is visible even though the oscillations are quite distorted, as is also reflected in their power spectra. These show that the strongest nodes, despite losing their harmonics, still have the most complex dynamics during anti-phase arrangement, with the largest peak at the synchronization frequency that is always lower than the natural. On the contrary, the weakest, unsynchronized nodes are speeding up compared to the isolated nodes for the LS and the Rössler models and are in general faster than the rest of the network, as is also visible in the time-series. It is also worth noting that the frequency depression is highest in the LS system, while chaotic oscillators are the least influenced by this.

### (iv) Pair-wise phase lags statistics.

**Figure 5.**
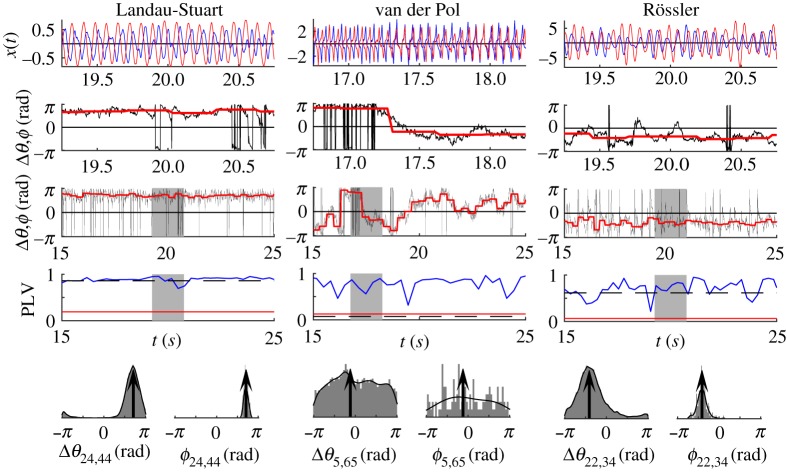
Time-series of the oscillatory neural activity at two regions, their PLV, phase lag and their statistics, for one inter-hemispheric link of LS, VdP and Rössler BNM. Top plots are the neural activity, and the two rows below them are the phase lags, Δ*θ*, (black), and angles of the cPLV, *ϕ*, (red for significant PLV, blue otherwise), where the upper plot shows the shaded part of the lower plot. Below them is PLV (blue) and the level of statistical significance (red), as well as the coherence over the whole time-series (dashed black). The bottom plots show the PDF (black) and histograms of phase lags, and their means (arrows). Nodes strengths are *K*_5_ = 0.24, *K*_22_ = 1.72, *K*_24_ = 3.91, *K*_34_ = 2.35, *K*_44_ = 5.63, *K*_65_ = 0.39 (maximum, minimum and mean of the connectome are 7.92, 0.19, 2.93). Parameters are the same as in figure 2 for frequency *f* = 20 Hz.

The most typical pair-wise correlation and phase coherence are illustrated in figure 5, and besides the in- and anti-phase synchronization, they include the varying case, when both are intermittently appearing. The left panel shows an intra-hemispheric link between in-phase synchronized brain regions, and the middle and the right panels depict inter-hemispheric links. In the first case, both nodes are weakly connected to the rest of the network, and hence epochs of in- and anti-phase locking exist, leading to multi-modal distribution of the phase lags. Unlike phase oscillators, when even the strongest nodes slip from anti- to in-phase arrangement, this only occurs between weak nodes for amplitude oscillators. As predicted by equation (2.7) [19], since *K*_24_ < *K*_44_ and the link is inter-hemispheric, the phase difference Δ*ϕ*_24,44_∈(*π*/2, *π*). On the other hand *K*_22_ < *K*_34_ and both nodes are in the left hemisphere, so Δ*ϕ*_22,34_∈( − *π*/2, 0). Finally, *K*_5_ < *K*_65_, so the inter-hemispheric phase difference should be Δ*ϕ*_5,65_∈(*π*/2, *π*) during the anti-phase epochs, as it is during the beginning of the highlighted interval, before slipping in ( − *π*/2, 0) for the rest of the interval when both regions are in-phase.

### (v) Whole-brain phase-lags statistics.

**Figure 6.**
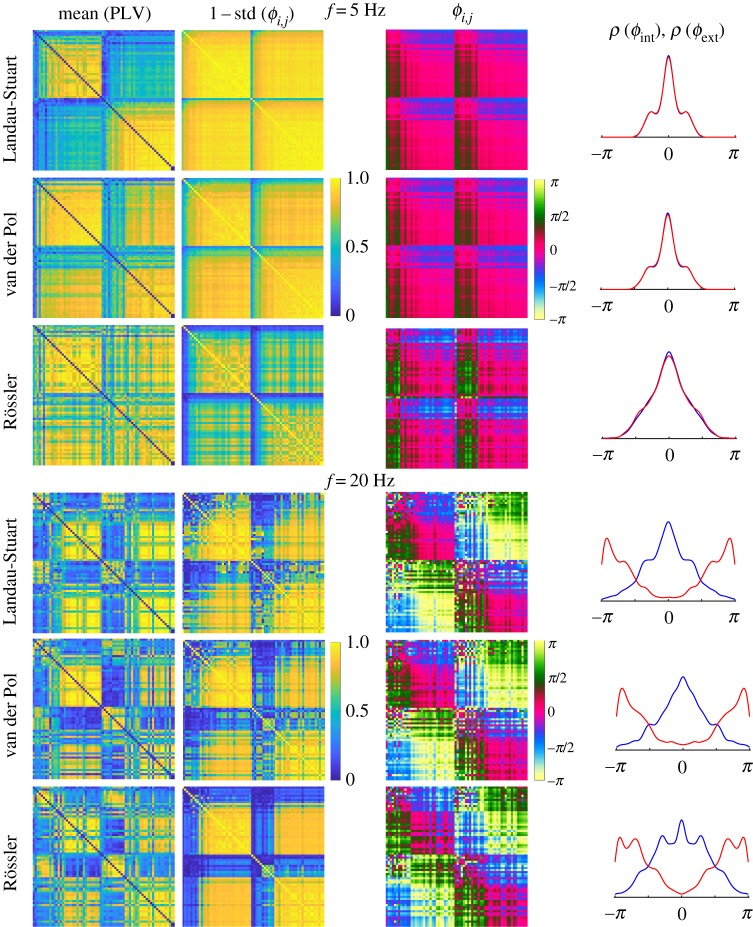
Statistics of PLV and phase lags for 68 brain regions ordered within hemispheres by the nodes strength. (left to right) Mean PLV, 1 − standard deviation of the phase lags and their mean for each link, and PDF of the mean phases for the internal (blue) and external (red) links. Parameters are the same as in figure 2.

The whole brain statistics for the distinctive phase regimes observed during oscillatory brain dynamics are shown in figure 6. It depicts the mean PLV for each pair of brain regions, and the correspondent mean and the standard deviation of the phase-lags calculated from cPLV. To keep the colours/coherence consistency across the images 1 − standard deviation is shown. All links in the plots have statistically significant coherence detected during at least five time windows, because the chosen method yields a low level of significance for PLV [19] (see also figure 5). The latter two metrics generally mirror the strength of the structural links and can be used to describe the functional connectivity, but are still differently affected by the underlying dynamics. For example, underrepresentation of the inter-hemispheric connections by the tracking techniques is clearly reflected in the PLV values for in-phase regimes, although lower levels of PLV do not necessarily invoke high variability of the phases. This is especially the case for the anti-phase dynamics of the chaotic BNM, but also the absence of intermittent in- and anti-phase dynamics for all the models, contrary to the Kuramoto oscillators [19], induces small spread of the phases due to their uni-modality, which is mainly affected by the strengths of the nodes. Thus, the high variance in figure 5 for the two weakest VdP oscillators is due to their very low overall phase coherence, despite their quite high average PLV.

The consistency of the regimes of synchronization, despite the weak level of statistical significance that renders all the links to be significantly coherent, still induces clearly pronounced peaks at 0 and ± *π* radians. The inter-hemispheric (external) links during the anti-phase regime are around ± *π* rad, contrary to the intra-hemispheric (internal) links that have always 0 centred phase lags, the same as the external links at low frequencies. Hence, the spatial distribution of phase-lags in figure 6 is in agreement with the theoretical predictions for Kuramoto oscillators, [19]. Moreover, strong regions lag behind the weaker, hence the green and blue shades for links in the phase lags matrices during in phase regimes. These get inverted for the anti-phase inter-hemispheric links, with darker shades corresponding to ± *π*/4 for internal links, and lighter for external with the values around *π* ± *π*/4.

## 4. Conclusion

In this paper, we computationally analyse the architecture of phases and amplitudes of large-scale neural oscillations, as they are shaped by the connectome. We identify exact features in the activity of the brain regions, given that the inherent neural dynamics is oscillatory and that the time delays due to the propagation in the connectome are proportional to the lengths of the structural pathways. In this way, we offer a framework for experimentally verifying the BNMs that utilize self-sustained oscillatory systems, which becomes more important in light of recent efforts towards building a probabilistic atlas of human cortical connections [52], specifically their time delays, which will eventually allow improving the utilized BNMs. In particular, the results can be used to test whether the inter-hemispheric time delays are significantly longer than the intra-hemispheric ones, which in the case of detectable coherence and large enough frequency should inevitably lead to anti-phase arrangement between some of the stronger-connected contralateral regions. In addition, we demonstrate that the delays necessarily reduce the activity at stronger regions for increasing frequency in linearly coupled oscillatory brain nodes.

We have extended previous studies of phases of brain regions that were derived from Kuramoto oscillators [19,28], and we have shown that the lagging of the stronger regions is universal for oscillatory BNMs, as has been experimentally observed [28,33]. The other consistent characteristic across the models is the decrease of the amplitude with an increase in the frequency and the coupling strength, especially pronounced for stronger nodes. This is in contrast to the findings about LS oscillators with time delays reduced to phase shifts [28], suggesting that besides the anti-phase locking, reduction of delays to phases also disregards other important aspects in the emerging dynamics. Furthermore, the amplitude reduction, as observed here, for the systems near the AF bifurcation is associated with a shift of the working point towards the bifurcation. This is the same mechanism responsible for the amplitude death [35,58], which for identical oscillators can be due to delayed interactions [59]. The amplitude death is amplified for distributed delays [60], such as those due to the connectome, but it is also increasing with the coupling strength, which for heterogeneous networks is stronger for stronger connected nodes.

With this study, we also confirm that phase reductions of time-delayed couplings, specifically the Kuramoto model, are sufficient for capturing the synchronization related aspects of the dynamics in networks of oscillators, especially those near AF bifurcation. Nevertheless, besides allowing for the study of the impact of the heterogeneous delays on the amplitude of the brain activity, increasing the dimensionality of the models as expected brings new peculiarities in the oscillatory dynamics. For instance, the phase-amplitude entanglement stabilizes the anti-phase ordering, which is otherwise sporadic for the delays of the connectome [19], while the frequency spectra become more complex due to nonlinearities. In addition, although delays can cause period doubling even for the mean-field of the KM [61], amplitude oscillators allow for coexistence of different types of local oscillations, such as chaotic, quasi-periodic and harmonic, within the same network. However, the current model assumes identical parameters for each region, and a more realistic approach would require this to be specified based on the data [23] or on some clinical hypothesis [25,62–64]. In this way, the specific characteristics of each region that translate in various spectral, isochronous or chaotic properties would also impact the overall dynamics, thus increasing the model's authenticity.

## Data accessibility

Data and codes are available at https://gitlab.thevirtualbrain.org/spase.petkoski/PhilTransRSocA2019.
